# The Effects of Polyphenol Supplementation in Addition to Calorie Restricted Diets and/or Physical Activity on Body Composition Parameters: A Systematic Review of Randomized Trials

**DOI:** 10.3389/fnut.2020.00084

**Published:** 2020-06-03

**Authors:** Fjorida Llaha, Raul Zamora-Ros

**Affiliations:** Unit of Nutrition and Cancer, Cancer Epidemiology Research Programme, Catalan Institute of Oncology (ICO), Bellvitge Biomedical Research Institute (IDIBELL), Barcelona, Spain

**Keywords:** polyphenol, supplements, physical activity, calorie restricted diet, body weight, fat, obesity, randomized clinical trials

## Abstract

**Background:** Both, calorie restricted diets (CRD) and physical activity (PA) are conventional obesity therapies but their effectiveness is usually limited in the long-term. Polyphenols are bioactive compounds that have shown to possess some anti-obesity properties. The synergic effects between dietary polyphenols and CRD or PA on body weight and fat are supported by several animal studies, but evidence in human is still inconsistent. Thus, our aim was to review the combined effects of polyphenol supplementation with CRD and/or PA on body weight and fat, body mass index (BMI) and waist circumference (WC) in overweight or obese adults.

**Methods:** Electronic databases (PubMed, Web of Science and Cochrane CENTRAL) were searched for randomized clinical trials (RCT) examining the combination of polyphenols with CRD and/or PA (up to December 31st, 2019). Articles were included if they had a duration of intervention ≥ 4 weeks. Both, quality and risk of bias of the included studies were assessed using the Cochrane RoB2 Tool.

**Results:** The review included 4 and 11 RCTs investigating the anti-obesity effects of polyphenol supplementation combined with CRD and PA, respectively. Isoflavone supplementation may increase fat loss during exercise among post-menopausal women in non-Asian studies. In the rest of RCTs regarding polyphenol supplementation and CRD or PA, no additive changes were found.

**Conclusion:** The results do not yet support polyphenol supplementation as a complementary strategy for enhancing the effectiveness of CRD and PA on weight and fat loss. However, this review suggests that isoflavone and soy products combined with lifestyle changes, especially exercise, provide additional anti-obesity effects in postmenopausal women. The potential role of polyphenols alone or, especially, in addition to conventional therapies (CRD and PA) mostly remains uncertain; and therefore, larger and longer RCTs examining these effects are needed.

**Protocol Registration:** PROSPERO CRD42020159890.

## Introduction

Decreasing body weight and fat lead to ameliorate obesity-related comorbidities, including diabetes mellitus (1), dyslipidemia (1, 2), hypertension (3, 4), cardiovascular diseases, and overall mortality (4). Calorie restricted diets (CRD) and the increase of physical activity (PA) are the conventional strategies recommended for obesity management (5). Attempts to sustain weight loss with CRD (6) and PA programs (7) induce compensatory biological and behavioral responses that difficult the maintenance of the reduced body weight over the long-term and usually cause weight regain after ending the treatment. Given the limitations of both CRD and PA, pharmaceutical and surgical approaches have been added to improve obesity treatments. However, the safety of pharmaceutical treatments in the long-term remains questionable as they may cause side effects (8). Moreover, surgical procedures, such as bariatric surgery, are invasive, expensive, and have their own inherent risks, including weight regain (9). The World Health Organization reported in 2016 that more than 1.3 billion and 650 million worldwide adults were overweight and obese, respectively (10). Therefore, safe, effective and simple alternative strategies for weight loss beyond the conventional ones, are extremely needed and have become a current hot topic in clinical and public health research.

Polyphenols are bioactive compounds ubiquitously found in plant-based foods and beverages such as tea, coffee, wine, fruits, vegetables, whole-grain cereals, and cocoa (11). They comprise a large variety of chemical structures which are divided into four main classes: flavonoids, and phenolic acids, lignans and stilbenes (12). A growing body of research indicates that polyphenols may reduce or maintain body weight. Indeed, after 5 years of follow-up, a significantly inverse association between polyphenol intake and body weight among 573 participants was observed in the PREDIMED (Prevención con Dieta Mediterránea) study (13). In a cross sectional study on 2,734 female twins, higher habitual intake of polyphenols was associated with a lower fat mass (14). The relation between different classes of polyphenols and weight loss have also been supported by several systematic reviews and meta-analysis of randomized clinical trials (RCTs) (15–17). Nevertheless, they generally agreed that further and larger studies are still needed to clarify the role of polyphenols in body weight and fat loss.

Overweight and obesity is caused by an imbalance between energy intake and energy expenditure. Weight and body fat loss can be usually achieved by reducing energy intake (following a CRD) or increasing energy expenditure (following a PA program) (5). Fat oxidation is the main pathway affected by PA (18). The metabolism responds to the reduction of energy intake by decreasing energy expenditure and decreasing fat oxidation in order to promote energy storage (6). Energy restriction affects also the neuro-hormonal system by decreasing anorexigenic hormone (e.g., leptin) and increasing appetite and orexigenic hormone (e.g., ghrelin) (6). The limitations of PA are more related to behavioral responses such as changes in eating behavioral, poor compliance to exercise programs and increase of the sedentary activities (7). Moreover, a decrease of the resting metabolic rate occurs during PA (7). Involvement of polyphenols in weight loss has been proposed due to their anti-obesity properties, such as: (i) stimulating thermogenesis and energy expenditure (19); (ii) inhibiting adipocyte differentiation and growth (20); (iii) increasing lipolysis and inducing β-oxidation (21); and (iv) decreasing appetite (22). It seems that polyphenols may mimic the PA pathways and strengthen the CRD changes, which led us to hypothesize that their combination may increase negative energy balance, increase fat oxidation, and provide a greater weight and fat loss.

Several animal studies have shown greater weight-lowering effects by adding polyphenol supplements to conventional strategies (23–25), but results from human studies are still inconsistent (26–28). However, many factors could affect the discrepancies between animal and human studies, such as: differences in the metabolism and mechanism of actions of polyphenols between animals and humans (29), and difficulties in controlling the weight, CRD and PA programs, and polyphenol intake in free living humans.

Evidences from human studies have indicated that polyphenol effects are significantly stronger after at least one month of intervention (30). The aim of the present study was to perform a systematic review of RCTs for investigating the potential effects of dietary polyphenol supplementation in addition to CRD and/or PA on body weight and fat, body mass index (BMI) and waist circumference (WC) changes in adults with overweight and obesity. Results on the additional effects of polyphenol supplementation on other obesity-related parameters were also discussed.

## Materials and Methods

### Search Strategy

**Figure 1:**
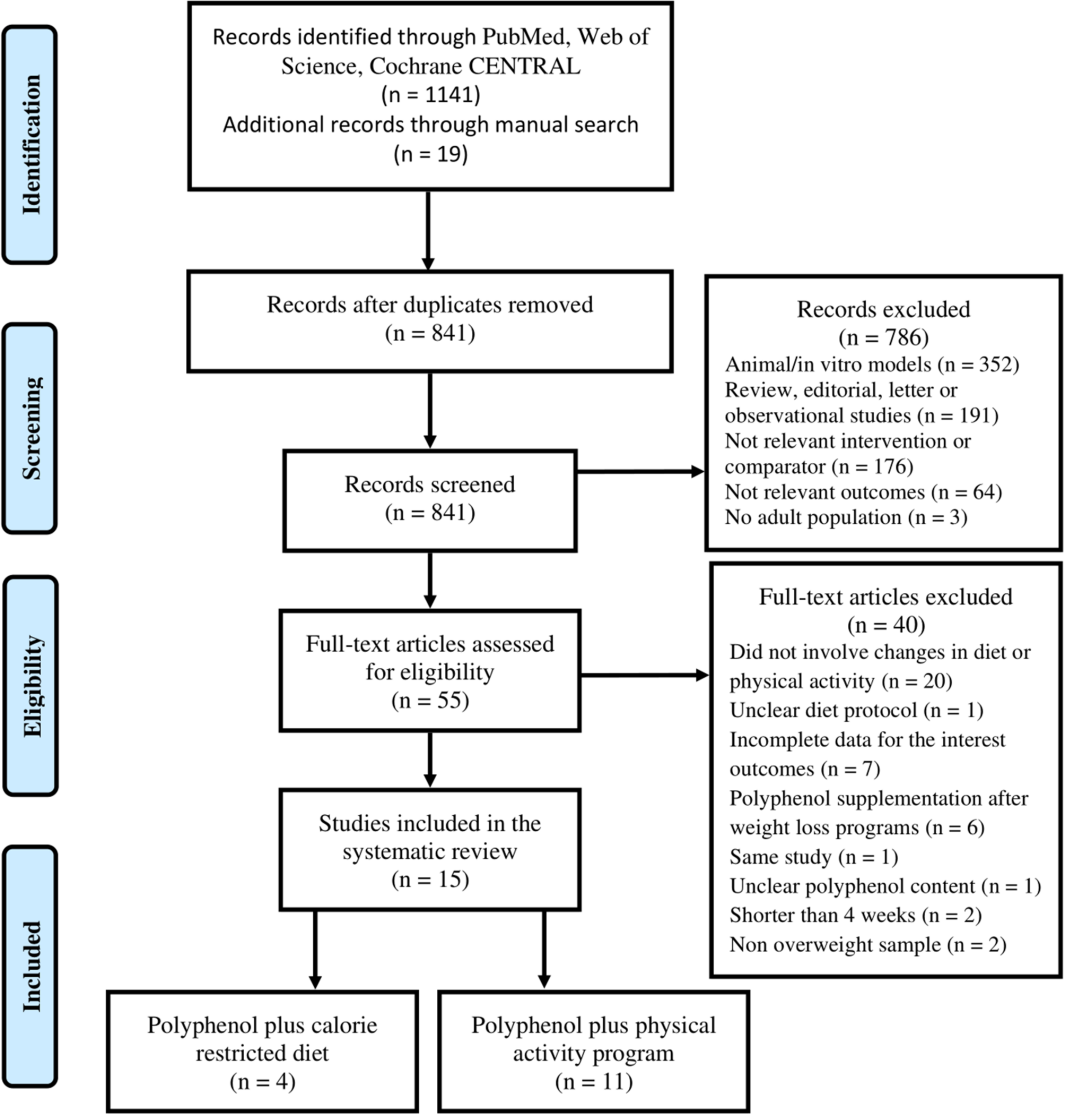
Study selection.

The review was designed according to the PRISMA guidelines. Our study protocol was previously registered in the PROSPERO database (CRD42020159890). To identify the articles, we searched in the following databases: PubMed, Web of Science, Cochrane CENTRAL up to December 31st, 2019. The search strategy was performed using these keywords: (“polyphenol” OR “flavonoid”) AND (“diet” OR “exercise” OR “training”) AND (“obesity” OR “body weight” OR “body fat” OR “waist circumference” OR “energy expenditure”). Reference lists of included manuscripts and relevant reviews were examined for any additional studies not previously identified (Figure 1). Both authors (FL and RZ-R) independently performed the search and the screen of the articles, and disagreements were discussed until consensus was reached. The research was limited to English language.

### Study Selection and Data Extraction

Eligible RCTs were selected for this review if they met the following criteria: (i) included adults with BMI ≥ 25 kg/m^2^; (ii) had a minimum of two groups of intervention: lifestyle alone (CRD and/or PA) and, lifestyle plus polyphenol supplementation: CRD plus polyphenol (CRD-PP) and/or PA plus polyphenol (PA-PP); (iii) reported at least two measurements (baseline and post-intervention) for body weight, BMI, WC, or body fat for the two groups of intervention (CRD and/or PA with and without polyphenols). Studies were excluded if they applied the polyphenol intervention after the weight loss program, reported incomplete data regarding CRD or PA programs (e.g., healthy Thai or Mediterranean diet, or enhanced physical activity), and did not clearly report the polyphenol supplementation content. When two publications were identified from the same study, we included only the most completed one regarding the body composition parameters and extracted data from both if it was necessary.

Both authors (FL and RZ-R) independently extracted data on the study design, country, participant characteristics, protocol of CRD and/or PA, polyphenol supplementation, outcome measures (e.g., body weight, BMI, WC, or body fat). In case of a multi-arm RCT occurred, outcome data were extracted only for the CRD and/or PA intervention groups with and without polyphenol supplementation. In addition, we extracted data from these studies about the effects of polyphenols on other obesity-related outcomes. Based on the heterogeneity and the limited number of studies, data was only summarized in a descriptive manner and was not meta-analyzed. In order to facilitate the synthesis and interpretation of the results, we have divided the studies by the type of the lifestyle intervention (CRD or PA).

### Risk of Bias in the Included Studies

The risk of bias of the included studies was independently assessed by both authors (FL and RZ-R) using the Cochrane RoB2 Tool (31). Disagreements were resolved by discussion until consensus was reached. Each domain was classified as low risk (fulfilling all criteria), medium risk (may have some issues that were likely to affect the conclusions), high risk (not fulfilling the criteria), and not available (insufficient details for judgment).

## Results

After screening of the eligible articles, 15 studies were included in this review. Four RCTs assessed the effects of polyphenol supplementation in addition to CRD (32–35), and 11 in addition to PA (26–28, 36–43). The reasons for the excluded articles are presented in Figure 1.

### Characteristics of the Included Studies

Two of the 4 studies that combined CRD with polyphenols were carried out in Spain, one in the United States and the last in the Netherlands. The number of participants ranged from 46 to 83. The polyphenol supplementation was combined with low calorie diets (daily restriction ≥ 500 kcal) during 1 to 3 months. The polyphenol supplementation varied between 81 and 1,125 mg/day and consisted in cocoa extract (1.4 g/day), epigallocatechin-gallate capsule, fresh grapefruit (384 g/day) or grapefruit juice (381 g/day), or green tea capsule. The RCTs were conducted among healthy adults or subjects without main cardiometabolic chronic diseases. Detailed characteristics of the studies are presented in Table 1.

**Table 1:** Characteristics of the studies that combined calorie restricted diets with polyphenols.

**References, Country**	**Length months**	**Age range (mean/SD)**	**% of females**	**N (CRD/ CRD-PP)**	**BMI by groups (mean/SD)**	**Health status**	**CRD Suppl**	**CRD-PP Suppl form: polyphenol type and dosage per day**	**Diet protocol**
Ibero-Baraibar et al. (32) Spain	1	50-80 (57.3/5.3)	54%	47 (24/23)	CRD: 30.3/1.9 CRD-PP: 30.7/2.5	Healthy	No Suppl	Cocoa extract: Flavanols 645 mg	LCD 15%^*^
Mielgo-Ayuso et al. (33) Spain	3	19-49	100%	83 (40/43)	CRD: 34.3/3.0 CRD-PP: 33.7/2.6	Healthy	Lactose Capsule	Capsule: EGCG 300 mg	LCD 600 Kcal^*^
Silver et al. (34) US	3	21-50 (38.7/8.2)	75%	68 (23/23 Gf/22 Gfj)	CRD: 35.7/3.5 CRD-PP (Gf): 36.3/3.1 CRD-PP (Gfj): 35.2/3.1	27% Metabolic Syndrome	Water	Gf: Naringin 81 mg Gfj: Naringin 119 mg	LCD 12.5%^*^
Diepvens et al. (35) Netherlands	3	19-57 (41.2)	100%	46 (23/23)	CRD: 27.6/1.8 CRD-PP: 27.7/1.8	Healthy	Md Capsule	GT-Capsule: Catechins 1125 mg (225 mg CF)	LCD

*BMI, body mass index (kg/m^2^); CF, caffeine; CRD, calorie restriction diet intervention group; CRD-PP, calorie restriction plus polyphenol intervention groups; EGCG, epigallocatechin-gallate; Gf, grapefruit; Gfj, grapefruit juice; GT, green tea; Kcal, kilo calories; LCD, low calorie diet; Md, maltodextrin; N, number of participants in both groups and (by groups); SD, standard deviation; Suppl, supplementation; US, United States*.

* *Calorie deficit per day*.

Three of the 11 studies that combined PA with polyphenols were carried out in Canada, three in Spain, two in Australia, one in United States, one in Iran and one in Brazil. The number of participants ranged from 33 to 107. The duration of the studies was between 3 and 24 months. Nine of the RCTs were done in healthy adults or without cardiometabolic chronic diseases. Only one RCT included subjects with insulin resistance (38), while another with non-alcoholic fatty liver disease (36). One Canadian RCT (40) combined an initial period of 6-months of isoflavone or placebo supplementation alone, with 6-months of isoflavone or placebo plus PA treatment. The effects of 40 to 100 mg/day of isoflavones provided by isoflavone capsules or soybean extract (*Glycine max*), were assessed by seven studies that recruited postmenopausal women only. Except one study that did not informed regard the isoflavones type, the others used an isoflavone mixture supplementation (genistein, daidzein, glycitein). Three of them administrated isoflavone supplementation rich in genistein (27, 37, 39), while other three, rich in daidzein (26, 28, 40). Three RCTs analyzed the effects of 300 to 625 mg/day of green tea catechins, while one investigated the effects of 500 mg/day of resveratrol. Participants of 8 studies completed an aerobic exercise or walking program, two a combined program of aerobic and resistance exercise, and one a resistance exercise program. Most of these studies applied a moderate-intensive PA program at heart rate between 60 and 75%, from 120 to 180 minutes/week. In addition to PA intervention, one RCT recommended to the participants to follow an energy-balanced diet (36). In one RCT, women were instructed to follow a 1,200 kcal/day diet (37). Also, two Spanish studies instructed their participants to adapt a Mediterranean diet pattern (27, 38). Detailed characteristics of the studies are presented in Table 2.

**Table 2:** Characteristics of the studies that combined physical activity with polyphenols.

**References, Country**	**Length months**	**Age range (mean/SD)**	**% of females**	**N (PA/ PA-PP)**	**BMI by groups (mean/SD)**	**Health status**	**PA Suppl**	**PA-PP Suppl form: polyphenol type and dosage per day**	**Physical activity protocol**
Barsalani et al. (26) Canada	6	50-70	100	39 (21/18)	PA: 29.75 (27.9-31.5)^*^ PA-PP: 30.29 (28.1-32.4)^*^	Healthy	Cellulose Capsule	Capsule: Isoflavone 70 mg (Daidzein 62.9%)	3 x 60 min/week AE^+^ RE, M-Int
Llaneza et al. (27) Spain	24	50-64 (56.7/3.5)	100	65 (32/33)	PA: 30.6/4.7 PA-PP: 30.5/4.2	Healthy	No Suppl	Glycine max: Isoflavone 80 mg (Genistein 76%)	5 x 30 min/week AE or walk
Choquete et al. (28) Canada	6	50-70 (58.7/5.3)	100	34 (18/16)	PA: 29.1/3.9 PA-PP: 30.2/3.5	Healthy	No Suppl	Capsule: Isoflavone 70 mg (Daidzein 62.9%)	3 x 60 min/week AE^.^ RE, M-Int
Llaneza et al. (37) Spain	6	50-64 (58.0)	100	70 (37/33)	PA: 35.2/4.78 PA- PP: 34.7/4.67	Healthy	No Suppl	Glycine max: Isoflavone 80 mg (Genistein 76%)	420 min/week Walk daily
Llaneza et al. (38) Spain	24	50-64 (56.2)	100	90 (44/46)	PA: 30.7/4.67 PA- PP: 29.6/4.23	IR	No Suppl	Capsule: Isoflavone 40 mg	420 min/week Walk daily
Orsatti et al. (39) Brazil	9	45-70 (56.2)	100	33 (18/15)	PA: 26.0/3.0 PA- PP: 30.3/4.7	Healthy	Lactose Capsule	Glycine max: Isoflavone 100 mg (Genistein 50%)	2 x 60 min/week RE, M-Int
Aubertin-Leheudre et al. (40) Canada	12	50-70 (58.0/5.0)	100	39 (18/21)	PA:30.0/2.0 PA- PP: 30.0/5.0	Healthy	Placebo Capsule	Capsule: Isoflavone 70 mg (Daidzein 62.9%)	3 x 60 min/week AE, M-Int
Hill et al. (41) Australia	3	45-70	100	38 (19/19)	PA: 31.39/0.73^+^ PA- PP: 30.65/0.59^+^	Healthy	Lactose Capsule	TEAVIGO: EGCG 300 mg	3 x 60 min/week Running, Int
Gahreman et al. (42) Australia	3	(26.0/0.7)^+^	0	43	PA:28.67/0.78^+^ PA- PP: 29.04/1.28^+^	Healthy	Placebo Capsule	GT Capsule: Catechins 562.5 mg (60 mg CF)	3 x 30 min/week AE, V-Int
Maki et al. (43) US	3	21-65 (48.0)	47.7	107 (51/56)	PA: 32.2/ 0.5^+^ PA-PP: 32.5/ 0.5^+^	Healthy	Placebo Beverage	GT: Catechins 625 mg (39 mg CF)	3 x 60 min/week M-Int
Faghihzadeh et al. (36) Iran	3	(45.2)	30	48 (24/24)	PA: 28.75/3.5 PA- PP: 28.35/3.49	NAFLD	Placebo Capsule	Capsule: Resveratrol 500 mg	3 x 3 0min/week M-Int

*AE, aerobic exercise; BMI, body mass index (kg/m^2^); CF, caffeine; EGCG, epigallocatechin-gallate; GT, green tea; Int, Intensive; IR, insulin resistance; N, number of participants in both groups and (by groups); NAFLD, non-alcoholic fatty liver disease; M-Int, moderate-intensive; PA, physical activity intervention group; PA-PP, physical activity plus polyphenols intervention group; RE, resistance exercise; SD, standard deviation; Suppl, supplementation; US, United States; V-Int, very-intensive*.

* *95% Confidence Interval*.

+ *Mean values with their standard error of the mean*.

### Effects of Polyphenols in Addition to Calorie Restricted Diets (CRD)

Body weight, BMI, WC, and body fat significantly decreased after both treatments: CRD and CRD-PP (Table 3). No differences were observed between both treatments (CRD vs. CRD-PP). A high dose of flavanols (1,125 mg/d) (35) did not provide greater results than lower doses (300 and 645 mg/d) (32, 33).

**Table 3:** Changes of the outcomes after the intervention with calorie restricted diets and polyphenols.

**Reference**	**Body weight (kg)**		**BMI (kg/m**^****2****^**)**		**WC (cm)**		**Body fat**		**Other outcomes**
	**Mean/SD**		**Mean /SD**		**Mean/SD**		**Mean/SD**		
	**CRD**	**CRD-PP**	**CRD**	**CRD-PP**	**CRD**	**CRD-PP**	**CRD**	**CRD-PP**	
Ibero-Baraibar et al. (32)	−2.5^*^	−2.7^*^	−0.9^*^	−1.0^*^	−4.3^*^	−5.2^*^	−1.3^*^ BF% −1.9^*^ TF%	−1.5^*^ BF% −2.74^*^ TF%	↔ glucose, insulin, LDL, HDL, TG ↓ oxLDL greater in CRD-PP
Mielgo-Ayuso et al. (33)	−7.7^*^	−7.6^*^	−3.0^*^	−3.0^*^	−4.0^*^	−5.0^*^	−4.6^*^ BF	−4.9^*^ BF	↔ fat oxidation, glucose, insulin, TC, HDL, LDL, CRP, RMR, IR.
Silver et al. (34)	−6.7^*^/ 3.1	Gf:−5.8^*^/3.1 Gfj:−5.9^*^/3.6	−2.1^*^ /1.1	Gf:−1.6^*^/1.6 Gfj:−1.9^*^/1.4	−5.4^*^ /4.8	Gf: - 4.0^*^/4.1 Gfj:−5.5^*^/4.7	−1.2^*^/2.6 BF% −1.2^*^/2.6 TF%	Gf:−1.1^*^/1.8 BF% Gfj:−1.1^*^/1.9 BF% Gf:−1.4^*^/2.9 TF% Gfj:−1.7^*^/2.6 TF%	↔ appetite, glucose, insulin, REE, RQ, IR, BP, TC, LDL, BLM. ^⊑^ HDL greater in Gfj.
Diepvens et al. (35)	−4.19^*^ /1.3	−4.21^*^ /2.7	−1.5^*^	−1.5^*^	−3.6^*^	−4.5^*^	−3.9^*^ BF	−3.8^*^ BF	↔ REE, RQ, SBP, DBP, HR. ^⊑^ appetite only in CRD-PP.

*BF, total body fat mass (kg); BF%, total body fat percentage; BLM, body lean mass; BMI, body mass index; BP, blood pressure; CRD, calorie restricted diet intervention group; CRD-PP, calorie restricted plus polyphenol intervention group(s); CRP, C-reactive protein; DBP, diastolic blood pressure; Gf, grapefruit; Gfj, grapefruit juice; HDL, high density lipoprotein cholesterol; HR, heart rate; IR, insulin resistance; LDL, low density lipoprotein cholesterol; oxLDL, oxidized low density lipoprotein cholesterol; REE, resting energy expenditure; RMR, resting metabolic rate; RQ, respiratory quotient; SBP, systolic blood pressure; SD, standard deviation; TC, total cholesterol; TF%, trunk fat percentage; WC, waist circumference*.

* *Significant changes within group (p ≤ 0.05)*.

↑ *Significant increase within group (p ≤ 0.05)*.

↓ *Significant decrease within group (p ≤ 0.05)*.

↔ *Not significant changes between groups (CRD-PP vs CRD, p > 0.05)*.

### Effects of Polyphenols in Addition to Physical Activity (PA)

Among the four studies that assessed the effects of isoflavone and reported data for body weight, the Canadian RCT (40) with 12 months of duration showed a significant weight loss after PA-PP treatment but not PA alone (Table 4). Additionally, a study that described the effects of isoflavone in different time-point and reported weight reduce at 6 and 12 months only in the group that received supplementation with isoflavone (data not shown) (38). Data regarding the effects of isoflavones on BMI were described by six RCTs. The Canadian RCT (40), revealed a BMI reduction only in the PA-PP group. Another Spanish RCT (27) with a 24-months duration showed a greater BMI decrease in the PA-PP compared to the PA group.

**Table 4:** Changes of the outcomes after the intervention with physical activity programs and polyphenols.

**References, Country**	**Body weight (kg)**		**BMI (kg/m**^****2****^**)**		**WC (cm)**		**Body fat**		**Other outcomes**
	**Mean (SEM)**		**Mean (SEM)**		**Mean (SEM)**		**Mean (SEM)**		
	**PA**	**PA-PP**	**PA**	**PA-PP**	**PA**	**PA-PP**	**PA**	**PA-PP**	
Barsalani et al. (26)	0.3	−1.5	0.0	−0.8	−5.1^*^	−6.6^*^	−2.4^*^ BF	−2.5^*^ BF	↔ BLM, glucose, insulin, IR, HDL, LDL. ↓ GGT, FLI (greater in PA-PP).
Llaneza et al. (27)			0.0	−2.6^*^	−0.6	2.0	5.3 BF	−4.8^*^ BF	↔ leptin, CRP, BLM, HDL, LDL, TC, TG. ↓ glucose, IR, TNF-a (greater in PA-PP).
Choquete et al. (28)	−0.8	−0.7	−0.3	−0.3	−5.5^*^	−5.4^*^	−1.0^*^ BF −0.5 TF	−1.7^*^ BF −0.8^*^ TF	↔ glucose, insulin, IR, LDL, HDL, TC. ↑ LMM only in PA-PP.
Llaneza et al. (37)			−0.6	−1.0	−0.9	−2.7	10.9 BF%	3.5 BF%	↔ BLM, insulin, IR, BP, HDL, LDL, CRP, TNF-a. ↑ adiponectin only in PA-PP.
Llaneza et al. (38)	−0.9	−0.2	−0.6	−0.2	−1.6	−0.1	−6.1 BF%	−4.3 BF%	↔ BLM, HDL, LDL, TG, TC, SBP, DBP. ↓ IR only in PA-PP.
Orsatti et al. (39)							0.8 BF −0.6 TF	0.7 BF −1.1 TF	↔ body muscle mass.
Aubertin-Leheudre et al. (40)	−1.0	−4.0^*^	0.0	−2.0^*^			−1.3 BF −0.39 AB	−2.9^*^ BF −1.9^*^ AB	↔ glucose, insulin, TC, LDL, HDL, TG, CRP. ↓ FFM/FM only in PA-PP
Hill et al. (41)	−0.45 (0.27)	0.08 (0.21)	−0.16 (0.1)	0.03 (0.08)	−2.69^*^ (0.55)	−1.02^*^ (0.64)	−0.8^*^ BF (0.2)	−0.2^*^ BF (0.3)	↔ BLM, glucose, leptin, adiponectin, lipids, CRP, BP, ↓ glucose in subjects with GI (greater in PA-PP).
Gahreman et al. (42)	−1.7^*^	−1.6^*^	−0.5^*^	−0.6^*^	−4.4^*^	−3.3^*^	−2.0^*^ BF −0.1^*^ AB	−2.3^*^ BF −0.2^*^ AB	↔ fat oxidation, BLM, RER, HR, VO2, glucose, LDL, HDL, TG.
Maki et al. (43)	−1.0^*^	−2.2^*^					−3.5^*^ BF% −0.3 AB%	−5.2^*^ BF% −7.7^*^ AB%	↔ LDL, HDL, TC. ↓ TG (greater in PA-PP).
Faghihzadeh et al. (36)	−1.1^*^	−0.9^*^	−0.4^*^	−0.4	−0.9^*^	−1.3^*^			↔ CRP, TNF-a, AST. ↓ ALT, IL-6 only in PA-PP. ↓ liver steatosis (greater in PA-PP).

*AB, abdominal fat mass (kg); AB%, abdominal fat percentage; ALT, alanine aminotransferase; AST, aspartate aminotransferase; BF, total body fat mass (kg); BF%, total body fat percentage; BLM, body lean mass; BMI, body mass index; BP, blood pressure; CRP, C-reactive protein; DBP, diastolic blood pressure; FFM/FM, fat free mass/fat mass ratio; FLI, fatty liver index; HDL, high density lipoprotein cholesterol; HR, heart rate; GGT, g-glutamyltransferase; GI, glucose intolerance; IL-6, interleukin-6; IR, insulin resistance; LDL, low density lipoprotein cholesterol; LMM, leg muscle mass; PA, physical activity intervention group; PA-PP, physical activity plus polyphenol intervention group(s); REE, resting energy expenditure; RER, respiratory energy rate; SBP, systolic blood pressure; SEM, standard error of the mean; TC, total cholesterol; TF, trunk fat mass (kg); TG, triglycerides; TNF-a, tumor necrosis factor alpha; WC, waist circumference;VO_2_, oxygen consumption peak*.

* *Significant changes within group (p ≤ 0.05)*.

+ *Mean changes in the indicated study are presented with their standard error of the mean*.

↑ *Significant increase within group (p ≤ 0.05)*.

↓ *Significant decrease within group (p ≤ 0.05)*.

↔ *Not significant changes between groups (PA-PP vs. PA, p > 0.05)*.

Isoflavone supplementation plus PA treatment but not PA alone caused a statistically significant total body fat mass loss in two of the RCTs (27, 40). Moreover, one study reported trunk fat mass loss only in PA-PP group but not in PA alone (28). Higher doses of isoflavones (100 mg/day) did not provide any extra effects on body composition parameters (39). The additional effects of them on fat loss were greater among participants who followed a 120–150 min/week moderate-intensive aerobic exercise or aerobic plus resistance than those doing resistance exercise alone (39). Participants that walked one hour daily, received isoflavone supplementation and were instructed to follow a diet of 1,200 kcal/day, showed the same results as those to the control group (walk and diet).

One study that combined 180 min/week of intensive running with epigallocatechin gallate supplementation during three months did not show any additional change in body composition parameters (41). Two studies that administrated green tea capsule and beverage with small amount of caffeine, did not reported greater results for weight, BMI, WC, and total body fat (42, 43). Green tea catechins with smaller amount of caffeine plus PA (39 mg) caused significant abdominal fat reduce, but not PA treatment alone (43). Resveratrol intervention combined with 90 min/week of moderate-intensive exercise and with the recommendation to follow an energy balanced diet did not provide any additional effects on BMI, WC, body weight, and fat reduce.

### Adverse Events of the Polyphenol Supplementation

Twelve of the fifteen selected studied informed about the adverse events due to polyphenols supplementation, while four did not do it (34, 40, 42). One case of hospitalization for high blood pressure was reported because of a supplementation with 500 ml/day of green tea (43). Seven participants that received 100 mg/day of isoflavones in a capsule, self-reported some slight discomforts in the gastrointestinal tract (39). No adverse events occurred in the rest of the RCTs.

### Risk of Bias in the Included Studies

Standardized risk of bias assessment was conducted following these domains: (i) randomization process; (ii) deviation from the intended intervention; (iii) missing outcome data; iv) measurement of the outcome; and (v) selection of the reported studies (Table 5). Three of the RCTs presented a low risk of bias in the five domains (32, 33, 36). Statement of randomization was reported, but the randomization method and allocation concealment were not specified in seven studies (28, 35, 37, 38, 40, 41, 43). However, differences between groups at baseline on these studies did not suggest a major problem with the randomization process. In any case, their bias due to the randomization process was classified at medium risk. Six RCTs did not perform a double-blind design, thus did not fulfill the low risk criteria of deviation from the intended intervention (27, 34, 37, 38, 41, 42). Only seven studies were classified at low risk of missing outcome data (28, 32–34, 36, 42, 43). Six of the RCTs did not fulfill the same criteria, mainly for two reasons: (i) the high rate of drop-outs or loss of follow-up and; (ii) the lack of an adequate analysis method that correct this bias (27, 37–41). The bias of missing outcome data was not assessed in two studies due to the lack of relevant information for judgment (26, 35). All studies used objective standardized body composition measures and assessed them properly, thus they were free of bias regarding the measurement of the outcomes. Selection of the reported results bias was also evaluated at low risk for all included studies.

**Table 5:** Risk of bias in the included studies.

**References**	**Randomization process**	**Deviations from intended interventions**	**Missing outcome data**	**Measurement of the outcome**	**Selection of the reported results**
Ibero-Baraibar et al. (32)	L	L	L	L	L
Mielgo-Ayuso et al. (33)	L	L	L	L	L
Silver et al. (34)	L	H	L	L	L
Diepvens et al. (35)	M	L	NA	L	L
Barsalani et al. (26)	L	L	NA	L	L
Llaneza et al. (27)	L	H	M	L	L
Choquete et al. (28)	M	L	L	L	L
Llaneza et al. (37)	M	H	M	L	L
Llaneza et al. (38)	M	H	M	L	L
Orsatti et al. (39)	L	L	H	L	L
Aubertin-Leheudre et al. (40)	M	L	M	L	L
Hill et al. (41)	M	M	M	L	L
Gahreman et al. (42)	L	M	L	L	L
Maki et al. (43)	M	L	L	L	L
Faghihzadeh et al. (36)	L	L	L	L	L

*L, low risk; M, medium risk (some concerns); H, high risk; NA, no available information for judgment*.

## Discussion

In this review, we have summarized the additional effects of polyphenol supplementation on body weight, BMI, WC, and body fat changes when combined with CRD and PA in adults with overweight or obesity. Comparing CRD or PA intervention groups with vs. without polyphenols helped to understand how polyphenols affect the efficacy of the CRD or PA on body composition parameters. The types of polyphenol supplementation were; isoflavone capsule and soybean extract, cocoa extract, grapefruit, and grapefruit juice, epigallocatechin gallate capsule, green tea capsule, and beverage, and resveratrol capsule. Isoflavone supplementation showed some additional effects in weight and fat loss during PA in overweight or obese postmenopausal women in the non-Asian studies. No additional effects were indicated for other types of polyphenols during CRD or PA. In addition, these RCTs investigated the effects of polyphenol supplementation on several cardiometabolic parameters related to obesity, showing some protective results on insulin resistance and inflammation markers.

Complementing CRD with one to three months of polyphenol supplementation did not provide any additional effect on weight and fat loss in overweight and obese adults. The results are consistent with findings from a previous review (17), showing that three months could be insufficient to detect significant polyphenol anti-obesity effects. CRD triggers adaptive responses by declining energy expenditure, which may persist for at least one year after the weight loss (44). Studies that assessed the effects of polyphenols after diet-induced weight loss found a prevention of weight regain by polyphenols (45, 46). These findings suggest that polyphenols might be more effective after the dynamic phase of the CRD in order to favor weight maintenance rather than for reducing weight per se during the CRD.

In comparison with CRD, the efficacy of PA was increased in some studies when polyphenols were added. It is important to bear in mind that the number of studies that assessed the effects of isoflavones in this review was larger and had longer duration (6 to 24 months). Particularly, mixture isoflavone supplementation (genistein, daidzein, glycitein) enhances the effects of PA (aerobic plus resistance exercise) on body composition parameters. Indeed, a higher loss of body weight and fat after PA plus isoflavone was observed compared to PA alone, in postmenopausal women of non-Asian studies (27, 40). Although, the mean weight loss of 1.5 kg in the isoflavone group in one of the RCTs (26) was not statistically significant, it is important to underline that 1 kg of weight loss is associated with a 16% reduction in diabetes risk (47). Isoflavones are flavonoids found mostly in soy products and are known as phytooestrogene due to their anti- and estrogenic properties. Adipose tissue express estrogen receptors, therefore, phytoestrogens may affect body composition directly by binding these receptors (48), then inhibiting lipogenesis and increasing lipolysis (49). Similarly to our findings, in a previous meta-analysis of RCTs phytoestrogens alone (including isoflavones) showed a significant decrease in body weight in healthy postmenopausal women that received isoflavone mixture supplementations (50). A subsequent meta-analysis in 2019 provided higher effectiveness of overall soy products in pre-menopausal women and in overweight or obese Asian participants (15). However, one Japanese RCT, which assessed the effects of isoflavone together with PA in postmenopausal women with BMI <25 kg/m^2^, reported no additional influence of isoflavone in body weight and fat; although it increases the body mineral density (51). The last meta-analysis (15) did not analyze the interaction between soy components and body weight which could have provided more insights into the weight-reducing role of each soy components: isoflavones, protein and fiber (15). Soy protein and fiber may confound the effectiveness of isoflavone by increasing satiety (52). Actually, an earlier meta-analysis that performed separate analysis for soy and isoflavones found anti-obesity effect of soy but not for isoflavone (53). Overall, it is difficult to establish the role of isoflavone in weight and fat loss because there are relevant differences between reviews (15, 50, 53) regarding the population ethnicity, menopausal and health status, and type and dose of isoflavone supplementation. A part from weight and fat loss, isoflavone supplementation showed improvement in liver function (26), inflammation (27, 37), and glycemia in women with insulin resistance (38). Although, two of the studies reported some beneficial effects in the fat free mass (28, 40), the majority did not observe any modification (26, 27, 37–39). Blood pressure and lipid profile of healthy women were not affected by isoflavones. Actually, the cardio-protective potential of isoflavone is stronger in persons with established hypertension (54) or hypercholesteremia (55).

Cocoa and its products (e.g., chocolate) are food sources rich in flavanols (catechins and proanthocyanidins). The addition of 1.4 g/day of cocoa extract to CRD during one month did not affect weight and fat loss compared to CRD alone (32). From animal studies, it has been suggested that the equivalent dose to a daily amount of 54 g of cocoa powder in human is necessary to have beneficial effects against obesity (56). Additionally, data from a meta-analysis of human studies that included all forms of cocoa/chocolate products, demonstrated that the intake of 30 g/day during 4–8 weeks caused a significant decrease on weight and BMI (30). No additional effects of cocoa occurred in glucose and insulin levels, but beneficial effects were noticed in oxidation status (32). Ibero-Baraibar and coworkers (57) also assessed the effects of cocoa in depression and found a decline of depressive symptoms only in the cocoa group. This is actually an important finding that could be considered in future cocoa-obesity-related research, as depression and obesity have a bidirectional relationship (58).

To our knowledge, few human studies have investigated the implication of grapefruit polyphenols on body weight and fat. Fresh grapefruit (384 g/day) and grapefruit juice (381 g/day) that provided 81 and 119 mg/day of naringin (flavanones) respectively, did not modify the anti-obesity potential of CRD treatment (34). These results are also in accordance with a meta-analysis of three RCTs that reported no influence of grapefruit on body weight (59). The effectiveness of a grapefruit capsule, juice and fruit supplementation on weight has shown to be greater in participants with metabolic syndrome compare to healthy subjects (60). Although, 27% of the participants in Silver et al. (34) had metabolic syndrome, the authors did not separate the analysis by it. Very high doses of grapefruit capsule intake (1,500 mg/day) has shown to cause some adverse events, particularly, gastrointestinal discomfort (60). The safety of different forms of grapefruit polyphenols intake at high doses deserves further investigation. In the RCT by Silver et al. (34), grapefruit and grapefruit juice did not confer additional activity in body lean mass, glycemia and blood pressure (34); whereas higher concentrations in serum HDL-cholesterol after CRD-PP treatment compared to CRD alone were observed (34). The results of the selected studies in this review were not supportive for any extra anti-obesity effects of green tea polyphenols (catechins) during CRD treatment. Diepvens et al. (35) explained that a possible reason of the weak thermogenic properties of green tea during a CRD could be the state of reduced sympathetic activity (reduced noradrenaline release). Dulloo et al. (61) observed that in the absence of increased noradrenaline release from sympathetic nerves, catechins, caffeine, or catechins plus caffeine had only mild effects on the thermogenesis of adipose tissue cells. Another potential explanation of the null effects of green tea could be the ethnicity of the participants of this review. It has been suggested that green tea may have greater influence in Asian rather than non-Asian participants (62) due to the genetic difference in the catechol O-methyltransferase (COMT) enzyme (63).

Green tea catechins inhibit COMT that degrades norepinephrine, which prolongs the action of sympathetically released norepinephrine, a key mediator to increase energy expenditure and promote the oxidation of fat (63). Caffeine intake is a potential co-factor that should be considered when analyzing green tea activity. In the RCT of Diepvens et al. (35) that included high caffeine consumers, no changes were reported by green tea. Similarly, evidence from a previous study demonstrated a stronger influence of green tea in obesity among habitual low caffeine consumers (<300 mg/day) compared to high caffeine consumers (≥300 mg/day) (45). The administration of green tea catechins without caffeine could not affect the anthropometric measures (64). Actually, the supplementation with 300 mg/day of epigallocatechin-gallate, the most abundant catechin in green tea, did not provide additional weight and fat loss during CRD treatment (33). The intervention of three months with epigallocatechin-gallate during PA treatment, also, did not result in an additional reduction of weight and fat (41). A meta-analysis of Kapoor et al. (65) reported an increase in metabolic rate even at low doses (300 mg/day) and suggested that epigallocatechin-gallate is an important moderator in fat metabolism. Thus, the relation of epigallocatechin-gallate and body weight, merit prospective research, especially long-term clinical trials. Among the two studies that administrated green tea that contained small amount of caffeine, 60 mg/day (42) and 39 mg/day (43), only the second one (43) that included subjects with higher BMI (>30 kg/m^2^) reported significant abdominal fat loss in PA-PP group but not PA alone. These discrepancies in results indicate that effects of green tea might be more evident in subjects with higher BMI. Incorporating green tea catechins in conventional strategies (CRD and PA) did not change their effects in glycemia (33, 41, 42), blood pressure (35, 41), lipid profile (33, 41, 42), and anti-inflammatory components (leptin, adiponectin and C-reactive protein) (33, 41). However, green tea showed a significantly greater decrease of glucose in persons with glucose intolerance (41), and a higher decline of triglycerides among participants with high triglyceride levels before the intervention (43). Body lean mass was not affected by green tea during PA treatment (41, 42). One study also reported no influence in exercise performance by considering the changes of oxygen consumption peak (VO_2_) (42). It is suggested that the improvement of exercise performance by green tea could be attributed, at least partly, to muscle glycogen sparing due to the stimulation of whole-body fat utilization (66). Therefore, this approach have been followed in physically fit subjects and showing an increase of whole-body fat utilization (67).

Resveratrol is a stilbene present in the skin of grapes, blueberries, raspberries as well as wine that can improve the metabolic syndrome (68). Three months of supplementation with 500 mg/day of resveratrol did not enhance the anti-obesity potential of PA in participants with a mean BMI = 28.5 kg/m^2^ and non-alcoholic fatty liver disease (36). Contrary, the same dose and period of intervention provided a decrease on anthropometric measures among adults with higher BMI (mean = 34.6 kg/m^2^) and metabolic syndrome (69). Indeed, a meta-analysis in 2019 indicated significant reductions on body weight, BMI and WC in obese participants at higher risk of metabolic disorders using resveratrol doses <500 mg/day in periods longer than three months (70). However, these results (70) presented a large heterogeneity regarding dosage and duration. Thus, further studies with established doses are warranted for a better comprehension of the anti-obesity potential of resveratrol. Although, weight and fat loss did not reach statistically significance in the Faghihzadeh et al. (36) study, resveratrol supplementation with PA provided more beneficial effects in liver function and inflammation compared to PA alone. Indeed, the current evidence is mostly supportive for the potential liver and cardio-protective effects of resveratrol (68).

## Strength and Limitations

Our review has some strengths. Firstly, this is the first review summarizing the additional effects of polyphenol supplementation in addition to a conventional obesity therapy (CRD and PA). Secondly, we did not restrict the study selection regarding the gender of participants, their health status, ethnicity, and type of polyphenol supplemented. We considered these differences during the interpretation of the results and a comprehensive understanding of the overall evidence was reached. However, some limitation should be also considered. The number of eligible and selected studies was small. Non-English studies were excluded, so we probably missed few studies, especially some Asian RCTs. Furthermore, the body composition parameters were not primary outcomes in all the included RCTs, and therefore, some data was missing. Due to incomplete data and the small number of studies, it was not feasible to perform a meta-analysis. Moreover, the included studies also presented some methodological drawbacks. Generally, they had a short duration, so the prolonged effects of polyphenols remain unclear. The small number of participants in most of the studies caused a low statistical power to identify significant differences. The compliance to polyphenol intake among the participants was uncertain in several of the studies. Only two RCTs (32, 39) measured it by plasma or urine metabolites, five RCTs counted the consumed containers (26, 34, 36, 42, 43) and eight did not report anything. Diet and PA outside the study protocol was not controlled in the majority of the RCTs. Moreover, some bias was detected regarding the randomization process, blindness and the missing outcome data bias. The methodological drawbacks should be considered by future researchers to minimize or avoid them.

## Conclusions

Our review suggests that combining isoflavone (70–80 mg/day) or soya products with conventional strategies, especially exercise, have potential beneficial effects in obesity management, particularly, in postmenopausal women. Findings from this review also suggest that the effects of polyphenols in metabolic parameters might be stronger in patients with already cardiometabolic diseases. Based on the current evidence, the anti-obesity potential of CRD and PA was not improved by adding other types of polyphenols. There is some evidence suggesting that polyphenols may be more effective in weight maintenance rather than inducing weight loss. For a better understanding of the influence of polyphenol supplementation during CRD and PA on body composition, further clinical trials with larger number of participants, longer duration (>12 months) and considering different polyphenol classes, doses and forms of administration are needed. Moreover, new RCTs should also focus on investigating the plausible implicated pathways to obesity, such as energy expenditure, fat metabolism, and appetite.

## Data Availability Statement

The original contributions presented in the study are included in the article/supplementary materials, further inquiries can be directed to the corresponding author/s.

## Author Contributions

FL and RZ-R contributed to conception and design, screening of the article, data extraction and assessing the quality of the studies. FL wrote the first draft and RZ-R critically revised and edited the manuscript. Both authors read and approved the final manuscript.

## Conflict of Interest

The authors declare that the research was conducted in the absence of any commercial or financial relationships that could be construed as a potential conflict of interest.

## Abbreviations

RCT: randomized clinical trial
CRD: calorie restricted diet
PA: physical activity
CRD-PP: calorie restricted diet plus polyphenol supplementation
PA-PP: physical activity plus polyphenol supplementation
WC: waist circumference.
